# Clinical and molecular determinants of response to maintenance olaparib for primary and recurrent epithelial ovarian carcinoma

**DOI:** 10.1007/s12094-025-04105-7

**Published:** 2025-11-20

**Authors:** Matthew K. Wagar, Kharmen Bharucha, Lauren Montemorano, Laura B. Huffman, Amy L. Godecker, Lisa M. Barroilhet

**Affiliations:** Division of Gynecologic Oncology, Department of Obstetrics and Gynecology, School of Medicine and Public Health, University of Wisconsin, 600 Highland Avenue, Madison, WI 53792 USA

**Keywords:** Ovarian cancer, PARP inhibitor, BRCA, Homologous recombination

## Abstract

**Purpose:**

To characterize long-term (LT) and short-term (ST) responders to the Poly (ADP-ribose) polymerase inhibitor olaparib in the primary and recurrent maintenance setting.

**Methods:**

Clinical and molecular data was collected for patients receiving maintenance olaparib between January 2014 and July 2021. ST responders were defined as those experiencing progression < 6 months from initiating olaparib, whereas LT responders exhibited response ≥ 2 years. Molecular analysis included germline *BRCA1/2* status, Myriad MyChoice CDx Homologous Recombination Deficiency (HRD) somatic testing, and STRATA Select comprehensive genomic profiling.

**Results:**

124 patients who received olaparib were included; 45 (36.3%) LT and 31 (25.0%) ST responders were identified. *BRCA2* mutations were enriched among the LT responders compared to ST responders (16 (35.5%) v. 4 (12.9%), *p* = 0.028) and LT responders were more likely to be HRD (40 (88.9%) v 19 (61.3%), *p* = 0.005). Patients receiving olaparib following platinum-sensitive recurrence were more likely to experience ST response compared to the primary setting (24 (55.8%) vs 7 (21.2%), *p* = 0.002). In both the primary and recurrent setting, LT responders were more likely to have a complete response to the most recent chemotherapy when compared to those experiencing ST response (24 (92.3%) v. 3 (42.9%), for primary maintenance, *p* = 0.021 and 10 (52.6%) v. 3 (12.5%) for recurrent, *p* = 0.004).

**Conclusions:**

LT response to olaparib for primary maintenance is common, and more frequently observed in patients with a complete response to platinum-based chemotherapy. *BRCA2* mutations conferred exceptional benefit to olaparib. Durable benefit was more commonly observed in patient’s receiving frontline maintenance olaparib.

## Introduction

Ovarian carcinoma remains the leading cause of death from gynecologic cancers, with over 12,000 estimated annual attributable deaths in the United States alone in 2024[1]. Primary treatment consists of cytoreductive surgery and platinum-based chemotherapy, however, following an initial response to therapy, approximately 70% of patients will experience relapse[2]. Given the high likelihood of recurrence, particularly for patients with advanced-stage disease, investigation into post-treatment maintenance therapies has expanded dramatically in the past decade. One such class of maintenance therapies are poly(ADP-ribose) polyermase (PARP) inhibitors. The PARP protein is involed in single-strand DNA repair, and inhibition of this mechanism can induce double-strand breaks in DNA[3]. This makes PARP inhibition particularly effective in homologus recombination deficient (HRD) tumors, exploiting the loss of this DNA repair pathway to trigger synthetic lethality in cancer cells[3]. Several recent prospective clinical trials have validated the benefits of PARP inhibitors as a maintenance strategy for epithelial ovarian cancer following response to platinum-based systemic therapy in both the primary and recurrent settings [4–9]. However, the most significant clinical benefit of PARP inhibitors is limited to patients with germline or somatic *BRCA* mutations and HRD ovarian cancers.

Despite integration into clinical practice, predictive biomarkers for sensitivity to PARP inhibitors remains limited to deleterious *BRCA 1/2* mutations and homologous recombination deficiency. Prior research has sought to characterize predictors of exceptional response (defined as progression-free survival ≥ 2 years) to PARP inhibitors following response to platinum-based systemic therapy in the recurrent setting, demonstrating that complete response to the most recent platinum-based chemotherapy regimen as the strongest clinical predictor of long-term response to PARP inhibition[10, 11]. Additionally, when patients are selected for PARP inhibitor maintenance on the basis of these biomarkers, approximately 10–20% of patients will experience progression within 6 months of beginning maintenance therapy[6, 8]. While post-hoc analyses from Study 19 [10]and ARIEL3 [11] both sought to characterize clinical and molecular data predictive of both short- and long-term response to PARP inhibitors in the recurrent setting, minimal data exist to predict exceptional response to PARP inhibitor maintenance therapy following primary treatment of ovarian carcinoma.

Given the ongoing incorporation of PARP inhibition into the frontline maintenance setting, predictive clinical and molecular biomarker data are needed[12, 13]. The purpose of this study was to identify and characterize the clinical and molecular profile of long-term (LT) and short-term (ST) responders to the PARP inhibitor olaparib following primary treatment of epithelial ovarian carcinoma. We secondarily assessed differences in determinants of response to olaparib across the primary and relapsed maintenance settings.

## Materials and methods

### Patient population

Patients with a diagnosis of epithelial ovarian carcinoma who received olaparib for maintenance therapy following platinum-based chemotherapy between January 2014 and July 2021 were identified retrospectively and included. All patients received olaparib for a minimum of 6 months unless disease progression was noted prior to this time point. Included patients were followed for a minimum of 24 months. Patients were excluded if they received olaparib for a therapeutic indication (ie for the treatment of ovarian malignancy without documented response to platinum prior to initiation) or received maintenance olaparib following receipt of a prior PARP inhibitor. This study was approved by the University of Wisconsin-Madison institutional review board (#2022-1691).

### Data collection

Clinical data were retrospectively collected and abstracted from the electronic medical record, including age at initiation of olaparib, Eastern Cooperative Oncology Group (ECOG) performance status, cancer stage, histology, timing of cytoreductive surgery (primary vs. interval), and presence of residual disease after cytoreduction. The number of prior chemotherapy regimens received, as well as the most recent response to platinum-based chemotherapy (partial vs. complete) was recorded. CA125 levels at diagnosis and the number of patients requiring dose modications while receiving olaparib were recorded.

Long-term (LT) response to olaparib was defined by a response of ≥ 2 years given current recommendations for maintenance therapy duration. Short-term (ST) response was defined as those experiencing disease progression < 6 months from initiating olaparib given the clinical implications of platinum resistance in this setting.

### Molecular testing

Assessment of germline mutational profile, including BRCA1/2 mutations, was performed using Myriad MyRisk testing (Myriad Genetic Laboratories, Salt Lake City, UT, USA), a next-generation sequencing panel of 48 cancer-related genes, including copy number variants. All genes of interest are analyzed for changes in coding regions and flanking introns. Somatic mutations were assessed using StrataNGS (Strata Oncology, Ann Arbor, MI, USA), a 429-gene polymerase chain reaction-based comprehensive genomic profiling test obtained from tumor tissue samples, including evaluation of single nucleotide variants, short insertions and deletions, copy number amplifications, deep deletions, gene fusions, microsatellite instability and tumor mutation burdern (TMB). HRD status was determined by Myriad MyChoice CDx Homologous Recombination Deficiency somatic testing and reported genomic instability scores (GIS). HRD status was defined by a GIS of ≥ 42 as determined by current clinical guidelines[14, 15] or the presence of a somatic or germline mutation in *BRCA1/2.* Classification of *TP53* mutations were dichotomized as gain of function (GOF) or loss of function (LOF) using the WHO International Agency for Research on Cancer Database. GOF mutations included: p.R175H, p.R248W, p.R248Q, p.R249S, p.R273H, p.R273L, and p.R282W.

### Statistical analysis

Medians, interquartile ranges (IQR), and proportions were calculated for continuous and categorial variables. Chi-square and Fisher’s exact tests were performed to assess categorical variables associated with LT and ST response to olaparib. Mann–Whitney and Kruskal–Wallis tests were performed to assess continuous factors associated with LT/ST response. Results were stratified by olaparib use in the primary or recurrent maintenance setting. Data analysis was performed using Stata Release 18 (StataCorp LLC, College Station, TX). A *p* value of < 0.05 was considered statistically significant. The data generated in this study are available upon request from the corresponding author.

## Results

### Clinical data

A total of 124 patients were identified who received olaparib maintenance therapy, of which 45 (36.3%) LT and 31 (25.0%) ST responders were noted. The majority of patients had cancers with serous histology (*n* = 117, 94.4%) and stage IIIC disease at presentation (*n* = 73, 58.9%). A greater proportion of patients experiencing LT response underwent optimal cytoreduction to microscopic disease compared to those experiencing ST response, although this was not statistically significant (93.3% v. 64.5%, *p* = 0.062). LT responders were more likely to have received one prior platinum-containing chemotherapy regimen compared to ST responders, who were more likely to have received two or more prior lines of platinum-based chemotherapy (57.8% v. 25.7%, *p* = 0.008). Additionally, LT responders to olaparib were more likely to experience a complete response to their most recent platinum-containing chemotherapy compared to ST responders (75.6% v. 22.5%, *p* < 0.001). Complete clinical characteristics are summarized in Table 1.

**Table 1:** Baseline Characteristics of long and short-term responders to Olaparib

	All others *n* = 48	LT *n* = 45	ST *n* = 31	*p* value
Age, median (IQR), years	62 (59–69)	65 (56–69)	64 (59–70)	0.644
ECOG				0.069
0	40 (83.3%)	42 (93.3%)	22 (70.9%)	
1	7 (14.6%)	3 (6.7%)	9 (29.1%)	
2	1 (2.1%)	0	0	
Histology				0.312
Serous	45 (93.7%)	43 (95.6%)	29 (93.5%)	
Endometrioid	1 (2.1%)	1 (2.2%)	0	
Carcinosarcoma	0	1 (2.2%)	2 (6.5%)	
Clear cell	2 (4.2%)	0	0	
Stage				0.063
IA	1 (2.1%)	0	1 (3.2%)	
IC2	0	1 (2.2%)	0	
IC3	1 (2.1%)	0	0	
IIA	2 (4.2%)	1 (2.2%)	0	
IIB	0	0	2 (6.5%)	
IIIA	3 (6.3%)	0	2 (6.5%)	
IIIB	2 (4.2%)	8 (17.8%)	1 (3.2%)	
IIIC	29 (60.4%)	23 (51.1%)	21 (67.6%)	
IVA	0	3 (6.7%)	2 (6.5%)	
IVB	10 (20.8%)	9 (20%)	2 (6.5%)	
Cytoreductive surgery				0.125
Primary	29 (60.4%)	30 (66.6%)	14 (45.2%)	
Interval	19 (39.6%)	15 (33.4%)	17 (54.8%)	
Disease after debulking				0.062
Microscopic	39 (86.6%)	42 (93.3%)	20 (64.5%)	
< 1 cm residual	7 (14.6%)	2 (4.5%)	7 (22.5%)	
> 1 cm residual	2 (4.2%)	1 (2.2%)	4 (13%)	
Prior chemotherapy regimens				**0.008**
1	27 (56.2%)	26 (57.8%)	8 (25.7%)	
2	18 (37.5%)	15 (33.4%)	14 (45.2%)	
3 or more	3 (6.3%)	4 (8.8%)	9 (29.1%)	
				** < 0.001**
Partial	22 (45.8%)	11 (24.4%)	24 (77.5%)	
Complete	26 (54.2%)	34 (75.6%)	7 (22.5%)	
CA125 median (IQR)	372 (209–1817)	584 (206–2357)	497 (157–1726)	0.8829
Dose modification	13 (27.1%)	10 (22.2%)	3 (9.8%)	0.173
Molecular testing				
* BRCAm*		33 (73.3%)	12 (38.7%)	**0.0025**
* BRCA1* mutant		17 (37.7%)	8 (25.8%)	0.275
* BRCA2* mutant		16 (35.5%)	4 (12.9%)	**0.028**
HR status				
HRD		40 (88.8%)	19 (61.3%)	**0.005**
HRP		5 (11.1%)	12 (38.7%)	
GIS score, median (IQR)		65 (48–75)	50 (43–65)	0.2227
*TP53*				0.371
Gain of function		6 (13.3%)	6 (19.3%)	
Loss of function		27 (60%)	15 (48.4%)	
HR pathway (non BRCA)		3 (6.6%)	4 (12.9%)	0.355
Cell cycle		7 (15.6%)	4 (12.9%)	0.747

Bold signifies statistical significance with a p-value <0.05

In total, 57 (46%) patients received olaparib as primary maintenance therapy and 67 (54%) patients received olaparib as recurrent maintenance therapy. There were no significant differences in age, ECOG, histology or stage at diagnosis between the primary and recurrent maintenance groups. A greater proportion of patients underwent interval cytoreduction in the group receiving olaparib maintenance therapy following primary treatment, compared to the recurrent setting (52.6% v. 31.3%). One patient who received olaparib maintenance therapy in the recurrent setting did not undergo cytoreductive surgery. Patients receiving primary olaparib maintenance were more likely to have experienced a complete response to platinum-based chemotherapy compared to those in the recurrent setting (84.2% v. 28.4%, *p* < 0.001). More patients experienced a ST response to olaparib when given for recurrent maintenance, compared to primary maintenance (35.8% vs. 12.3%, *p* = 0.002). Characteristics according to receipt of primary or recurrent maintenance olaparib are noted in Table 2.

**Table 2:** Characteristics of patients receiving Olaparib for primary vs. recurrent maintenance therapy

	Primary *n* = 57 (46%)	Recurrent *n* = 67 (54%)	*p* value
Age, median (IQR), years	63 (57–68)	64 (59–70)	0.3655
ECOG			
0	52 (91.2%)	52 (77.6%)	0.106
1	5 (8.8%)	14 (20.8%)	
2	0	1 (1.6%)	
Histology			0.393
Serous	52 (91.2%)	65 (96.8%)	
Endometrioid	1 (1.8%)	1 (1.6%)	
Clear cell	2 (3.5%)	0	
Carcinosarcoma	2 (3.5%)	1 (1.6%)	
Stage			0.199
IA	0	2 (3.2%)	
IC2	0	1 (1.6%)	
IC3	0	1 (1.6%)	
IIA	1 (1.8%)	2 (3.2%)	
IIB	0	2 (3.2%)	
IIIA	2 (3.5%)	3 (4.4%)	
IIIB	6	5 (7.4%)	
IIIC	30	43 (63.3%)	
IVA	3	2 (3.2%)	
IVB	15	6 (8.9%)	
Cytoreductive surgery			
Primary	27 (47.4%)	45 (67.2%)	**0.019**
Interval	30 (52.6%)	21 (31.3%)	
Disease after debulking			0.962
Microscopic	47 (82.4%)	54 (80.6%)	
< 1 cm residual	7 (12.3%)	9 (13.4%)	
> 1 cm residual	3 (5.3%)	3 (4.5%)	
Prior chemotherapy regimens			
1	57 (100%)	5 (7.5%)	** < 0.001**
2	0	47 (70.1%)	
3 or more	0	15 (22.4%)	
Response to most recent chemotherapy			** < 0.001**
Partial	9 (15.8%)	48 (71.6%)	
Complete	48 (84.2%)	19 (28.4%)	
Response to olaparib			**0.002**
LT	26 (45.6%)	19 (28.4%)	
ST	7 (12.3%)	24 (35.8%)	
Clinical trial participation	9 (15.7%)	7 (10.4%)	0.291

Bold signifies statistical significance with a p-value <0.05

In the primary maintenance group, 26 patients (45.6%) experienced an LT response while seven (12.3%) experienced an ST response (Table 3). There were no significant differences in age, histology, stage at diagnosis of presence of microscopic vs. macroscopic disease after cytoreductive surgery between the LT and ST groups who received primary olaparib maintenance. Notably, all patients experiencing ST response underwent primary cytoreductive surgery. More patients experiencing LT response to primary olaparib maintenance experienced a complete response to platinum-based chemotherapy compared to those experiencing ST response (24, 92.4% v. 3, 42.9%, *p* = 0.021). In the recurrent maintenance group, 19 patients experienced an LT response while 24 experienced an ST response (Table 4). There were no significant differences in age, ECOG, histology, stage at diagnosis, receipt of secondary debulking or number of prior platinum-containing chemotherapy regimens between the LT and ST groups who received recurrent maintenance therapy. All patients experiencing LT response to recurrent olaparib maintenance underwent optimal cytoreduction to microscopic disease (*p* = 0.031). LT responders demonstrated a significantly longer platinum-free interval compared to ST responders (20 months (IQR 12–36) v. 11 months (IQR 7–18) *p* = 0.0098). ST responders were more likely to have had a partial response to the most recent platinum-containing chemotherapy, compared to LT responders in the recurrent maintenance group (21 (87.5%) v. 9 (47.4%), *p* = 0.004).

**Table 3:** Characteristics of Long and Short-term responders receiving Olaparib as primary maintenance *n* = 33

	LT *n* = 26	ST *n* = 7	*p* value
Age, median (IQR), years	64 (56–68)	66 (61–82)	0.1343
Histology			0.525
Serous	24	6	
Endometrioid	1	0	
Carcinosarcoma	1	1	
Stage			
IIA	1	0	0.263
IIIB	4	0	
IIIC	10	6	
IVA	3	0	
IVB	8	1	
Cytoreductive surgery			
Primary	14	7	**0.011**
Interval	12	0	
Disease after debulking			0.162
Microscopic	23	4	
< 1 cm residual	2	1	
> 1 cm residual	1	2	
Response to most recent chemotherapy			**0.021**
Partial	2 (7.7%)	4 (57.1%)	
Complete	24 (92.3%)	3 (42.9%)	
		45.5%)	

Bold signifies statistical significance with a p-value <0.05

**Table 4:** Characteristics of long and short-term responders receiving Olaparib as recurrent maintenance n = 43

	LT *n* = 19	ST *n* = 24	*p* value
Age, median (IQR), years	66 (56–71)	64 (57–69)	0.5486
ECOG			
0	17 (89.5%)	17 (70.8%)	0.136
1	2 (10.5%)	7 (29.2%)	
Histology			0.368
Serous	19 (100%)	23 (95.8%)	
Carcinosarcoma	0	1 (4.2%)	
Stage			0.219
IA	0	1 (4.2%)	
IC2	1 (5.2%)	0	
IIB	0	2 (8.3%)	
IIIA	0	2 (8.3%)	
IIIB	4 (21.1%)	1 (4.2%)	
IIIC	13 (68.5%)	15 (62.5%)	
IVA	0	2 (8.3%)	
IVB	1 (5.2%)	1 (4.2%)	
Cytoreductive surgery			0.053
Primary	16 (84.2%)	14 (58.3%)	
Interval	3 (15.8%)	10 (41.7%)	
Disease after debulking			**0.031**
Microscopic	19 (100%)	16 (66.6%)	
< 1 cm residual	0	5 (20.8%)	
> 1 cm residual	0	3 (12.6%)	
Prior chemotherapy regimens			0.244
1	0	2 (8.3%)	
2	15 (78.9%)	14 (58.3%)	
3 or more	4 (21.1%)	8 (33.4%)	
Response to most recent chemotherapy			**0.004**
Partial	9 (47.4%)	21 (87.5%)	
Complete	10 (52.6%)	3 (12.5%)	
Platinum Free Interval, median (IQR), mo	20 (12–36)	11 (7–18)	**0.0098**
Secondary cytoreduction	5 (26.3%)	4 (16.6%)	0.44

Bold signifies statistical significance with a p-value <0.05

### Molecular analysis

**Fig. 1:**
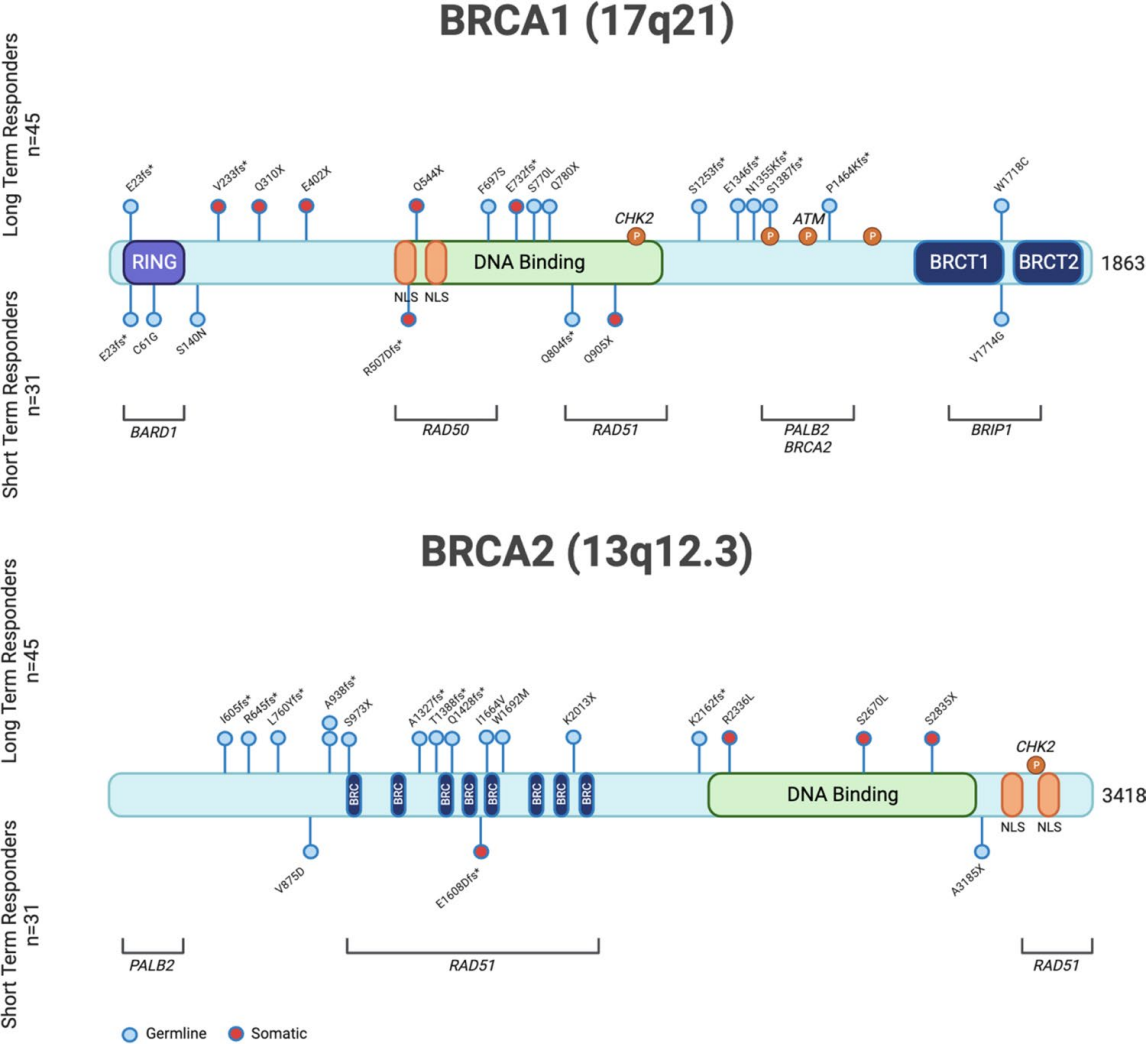
Location of *BRCA 1/2* mutations along the *BRCA 1/2* genes for patients receiving olaparib maintenance. Two deep deletions in *BRCA1* LT group not shown. *BRCA1* splice site mutation in ST group not shown. Large somatic rearrangement of *BRCA2* found in ST group not shown

33 (73.3%) of LT responders demonstrated germline or somatic mutations in *BRCA1/2* compared to 12 (38.7%) of ST responders (*p* = 0.0025) (Table 1). A greater number of *BRCA2* mutations were found in LT responders compared to ST responders (35.5% vs 12.9%, *p* = 0.028), while there was no significant difference in *BRCA1* mutations between the LT and ST groups (*p* = 0.275). We further characterized the location of mutations in BRCA1/2 (Fig. 1). Of the 17 LT responders with *BRCA1* mutations, 12 patients had *BRCA1* germline mutations where five patients had *BRCA1* somatic mutations. Of the 16 LT responders with *BRCA2* mutations, 12 patients had *BRCA2* germline mutations and 4 had *BRCA2* somatic mutations. In the ST responders, seven patients had germline mutations in *BRCA1*, while one patient had a somatic mutation in *BRCA1*. Two ST response patients had germline *BRCA2* mutations, while two patients had somatic *BRCA2* mutations. Mutations in the *RAD51* domain (*n* = 7) and DNA binding domain (*n* = 3) of *BRCA2* were more frequently observed in those with LT response compared to ST (Fig. 1).

LT responders were more likely to have HRD (88.8%) compared to ST responders (61.2%, *p* = 0.005). No significant difference was seen between LT and ST responders according to the median GIS score (Table 1). Of the 19 patients with HRD in the ST responder group, 7 were *BRCA*wt.

**Fig. 2:**
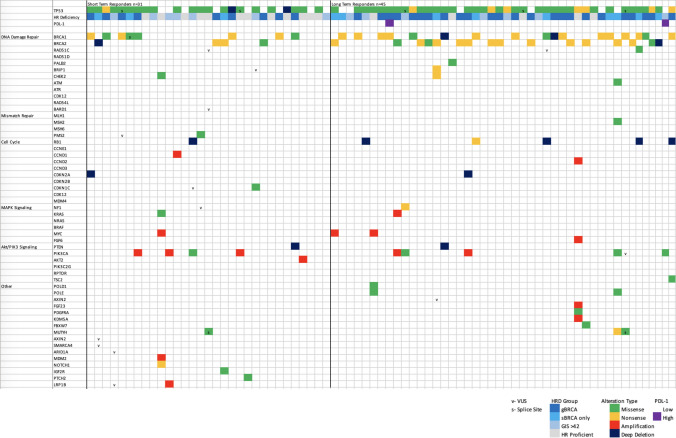
Mutations and gene alterations in LT and ST responders to maintenance olaparib

A total of 160 likely functional genetic alterations in 55 genes were found, with *TP53* (78.9%), *BRCA1* (32.9%), and B*RCA2* (26.3%) being the most common (Fig. 2). TP53 mutations were noted in the majority of LT (73.3%) and ST (67.7%) responders. There were no significant differences in the proportion of patients with TP53 gain-of-function mutations compared between the LT and ST groups (*p* = 0.2227). Other types of gene mutations and amplifications were observed affecting the MAPK signaling pathway, PIK3 signaling and cell cycle regulation (Fig. 2). Notably, six mutations in RB1 were noted, 5 of which were amongst the LT responder group (4 deep deletions, 1 nonsense mutation). There were no significant differences in the prevalence of non-BRCA HR pathway mutations (*p* = 0.355) or cell cycle gene mutations (*p* = 0.747) between the LT and ST responders.

## Discussion

Following receipt of platinum-based chemotherapy, patients with epithelial ovarian carcinoma who experienced a complete response to platinum in the primary setting were more likely to experience ≥ 2 years of benefit from maintenance therapy with olaparib. 36.3% of patients experienced LT response to olaparib, with 46.3% of patients experiencing LT response when olaparib was administered as primary maintenance. Mutations in *BRCA1/2* were associated with LT response to olaparib, with mutations in BRCA2 enriched among the LT responders.

Our analysis adds to prior research examining clinical predictors of response to maintenance olaparib, which has previously been completed largely in the recurrent setting. Lheureux et al. conducted a comparative clinical and molecular analysis of pooled data from the phase 2 Study 19 and Study 41 trials, both of which examined olaparib maintenance for relapsed platinum sensitive ovarian cancer [10, 16, 17]. Of 217 patients in the combined cohort who received olaparib maintenance, 52 (23.5%) experienced exceptional response defined as > 2 years without evidence of disease progression. Similar to our findings, LT response was associated with complete response to the most recent chemotherapy (*p* < 0.05). Our findings demonstrate a similar proportion of patients experiencing LT response in the recurrent setting (28.3%), however, 45.6% of patients in the primary olaparib group experienced LT response to maintenance therapy, demonstrating exceptional durable benefit for these patients when received as a component of primary therapy. Based on our current results, a complete response to the most recent platinum therapy, particularly in the primary setting was associated with LT response to olaparib maintenance. Platinum sensitivity remains a strong clinical predictor of PARP inhibitor efficacy. O’Malley et al. in an exploratory analysis of the phase 3 ARIEL3 trial, revealed no measurable disease following most recent treatment, response to latest platinum and longer platinum free interval as correlates to exceptional benefit with the PARP inhibitor rucaparib [11]. Our results are consistent with this existing data and report a significantly greater proportion of LT responders experiencing a complete response to their most recent platinum based chemotherapy in both the primary (92.3%) and recurrent groups (52.6%). It is worth noting that all ST responders in the primary maintenance group underwent primary cytoreduction, suggesting that for patients receiving adjuvant platinum therapy following surgical debulking, lack of radiographic measurable disease response may pose a challenge for risk-stratifying these patients based on clinical factors alone.

Molecular data describing patients who experience durable benefit to PARP inhibitor maintenance is limited to those who experience relapse. Both germline and somatic mutations in *BRCA1/2* were associated with LT response to olaparib, with a particular enrichment of *BRCA2* mutations in the LT response group. Our findings are consistent with prior data suggesting differences in response to platinum-based therapies and outcomes between *BRCA1* and *BRCA2* genotypes[10, 18, 19]. In a 2011 observational study of clinical and genomic data from the cancer genome atlas project, Yang et al. reported a significantly improved overall survival (0.33; 95% CI 0.16–0.69; *P* = 0.003) for patients with *BRCA2* mutations compared to *BRCA*wt, which was not observed in BRCA1-mutated cases. Furthermore, *BRCA2* mutations were associated with higher primary chemotherapy sensitivity (100% vs 80%, p = 0.05), as well as longer platinum-free intervals (18 months vs 12.5, *p* = 0.04), compared to patients with *BRCA1* mutations[18]. Many LT responders in our cohort demonstrated germline mutations in the *RAD51* and DNA-binding domain of BRCA2. The RAD51 binding domain is a frequent site of mutations in BRCA2, and mutations in this region impair the ability to load RAD51 to sites of DNA-damage [20]. Recently, RAD51 has emerged as a biomarker for platinum sensitivity. Compadre et al. evaluated platinum responsiveness using immunofluorescence in high-grade serous ovarian cancer patients via a novel RAD51 score based on the presence of RAD51 foci[21]. Tumors demonstrating low RAD51 scores (those that cannot form RAD51 foci, and therefore, cannot use homologous recombination to repair DNA, leading to synthetic lethality) demonstrated a high degree of platinum sensitivity, and in a validation cohort, a low RAD51 score demonstrated 100% positive predictive value for platinum sensitivity. Our data support these findings and suggest that interventions preventing loading of RAD51 by BRCA2 could potentiate platinum sensitivity.

Our analysis does not identify a specific mechanism involved in the 26.6% of patients with *BRCA1/2wt* genotypes experiencing LT response to olaparib. The majority of patients experiencing LT response were HRD (88.8%), reflecting current indications for PARP inhibitor maintenance in the primary and relapsed settings. Of the five patients with HRP phenotypes demonstrating LT response, all harbored a mutation in *TP53*; one possessed a *MYC* amplification, as well as mutations in *POLE* and *POLD1*. Another demonstrated a mutation in *PALB2*, while another demonstrated several mutations in across the cell cycle and MAP kinase pathways among others including: *CCND2, FGF6, FGF23, PDGFRA*, and *KDM5A*. The remaining two patients both demonstrated no mutations outside of their *TP53* mutations. Four patients demonstrating mutations in *RB1* (3 deep deletions, 1 nonsense mutation) experienced LT response to olaparib. Prior evidence suggests exceptional survival when *RB1* deletions co-occur in the presence of *BRCA1/2* mutations, which was the case for three of the four LT responders with *RB1* mutations identified[22]. Garsed et al. characterized 96 samples from a group of ovarian cancer patients surviving > 10 years past diagnosis obtained from the gynaecological oncology biobank at Westmead Hospital using immunohistochemistry and genome sequencing. Of those undergoing whole genome sequencing, 54.5% were found to have inactivation of *RB1*. However, these results are unable to be characterized in the era of PARP inhibitor maintenance, and the utility of PARP inhibitors in enhancing this survival observation is uncertain.

While the majority of patient’s in the present study harbored a mutation in *TP53*, there was no significant difference in the presence of GOF mutations between LT and ST responders. Prior research has implicated gain-of-function mutations in *TP53* as carrying a worse prognosis and a higher incidence of platinum resistance compared to mutations contributing to LOF[23]. The low number of GOF mutations observed in our cohort makes these results difficult to interpret with sequencing data alone and further studies may benefit incorporating a broader array of molecular testing to elucidate the role of *TP53* in platinum response in the era of PARP inhibitors.

The utilization of PARP inhibitors as a maintenance therapy in ovarian cancer has been steadily increasing over the past decade, particularly in the front-line setting[24]. Given recent changes to PARP inhibitor indications, an understanding of factors contributing to exceptional benefit from these maintenance medications is crucial to adapting use to a changing clinical landscape. As more patients receive PARP inhibitors in the frontline setting, selection of patients with a response to platinum-based chemotherapy remains a strong predictor of benefit to PARP inhibitor maintenance.

A strength of our study is the availability of complete follow-up data for all included patients for a minimum of 2 years. Additionally, the majority of patients identified in our comparison of LT and ST responders met current criteria for PARP inhibitor maintenance based on the presence of BRCA1/2 mutations and HRD status, making our results applicable to contemporary clinical populations. Our study is limited by the use of tissue samples which were obtained at varying clinical points depending on the patient’s clinical presentation. As such, the standardization of our samples is not uniform across primary cytoreductive surgery, interval cytoreduction or biopsy obtained at primary diagnosis or relapse. We are unable to make conclusions regarding dynamic genetic changes that may occur from primary diagnosis to when relapse occurs, and further evaluation incorporating temporal changes would be beneficial. Our study focused on predictors of response to olaparib in the primary setting, and future studies should focus on dynamic biomarkers capable of identifying when maintenance therapy may no longer provide ongoing benefit. Additionally, we did not capture data regarding time to second progression or other metrics that would characterize response of subsequent therapies after progression on olaparib maintenance, which is an area of active investigation.

Our results demonstrate that LT response to olaparib in the primary maintenance setting is common, and more frequently observed in patients with favorable clinical characteristics, such as platinum sensitivity. Both germline and somatic *BRCA2* mutations conferred exceptional benefit to olaparib maintenance, and an understanding of the interaction with other HRD-related genes, such as *RAD51,* could lead to novel biomarkers and therapeutics for those who would otherwise not receive maintenance therapies. Durable benefit was more commonly observed in patient’s receiving frontline maintenance olaparib. As indications for PARP inhibitors move these medications to the primary setting, future studies aiming to identify additional predictors of exceptional response, and characterize the multifactorial molecular contributors to this observed response are warranted.

## Data Availability

Not applicable.
